# *Saccharomyces cerevisiae* additions normalized hemocyte differential genes expression and regulated crayfish (*Procambarus clarkii*) oxidative damage under cadmium stress

**DOI:** 10.1038/s41598-023-47323-1

**Published:** 2023-11-28

**Authors:** Yaru Yang, Shuaidong Li, Yumin Zhu, Litao Che, Qifan Wu, Shijun Bai, Guocheng Shu, Xianming Zhao, Peng Guo, Salma A. Soaud, Nianzhen Li, Mengling Deng, Jia Li, Ahmed H. El-Sappah

**Affiliations:** 1 School of Agriculture, Forestry and Food Engineering, Yibin University, Yibin, 644000 China; 2 College of Morden Agriculture, Yibin Vocational and Technical College, Yibin, 644003 China; 3 Genetics Department, Faculty of Agriculture, Zagazig University, Zagazig, 44511 Egypt; 4 Faculty of Animal Science and Technology, Yunnan Agricultural University, Kunming, Yunnan 650201 China

**Keywords:** Genetics, Microbiology, Molecular biology, Environmental sciences

## Abstract

Because China produces the most crayfish in the world, safe solutions must be improved to mitigate the risks of ongoing heavy metal stressors accumulation. This study aimed to use *Saccharomyces cerevisiae* as a bioremediation agent to counteract the harmful effect of cadmium (Cd) on crayfish (*Procambarus clarkia*). Our study used three concentrations of *S. cerevisiae* on crayfish feed to assess their Cd toxicity remediation effect by measuring total antioxidant capacity (TAC) and the biomarkers related to oxidative stress like malondialdehyde (MDA), protein carbonyl derivates (PCO), and DNA–protein crosslink (DPC). A graphite furnace atomic absorption spectroscopy device was used to determine Cd contents in crayfish. Furthermore, the mRNA expression levels of lysozyme (*LSZ*), metallothionein (*MT*), and prophenoloxidase (*proPO*) were evaluated before and following the addition of *S. cerevisiae*. The results indicated that *S. cerevisae* at 5% supplemented in fundamental feed exhibited the best removal effect, and Cd removal rates at days 4th, 8th, 12th, and 21st were 12, 19, 29.7, and 66.45%, respectively, which were significantly higher than the basal diet of crayfish. The addition of *S. cerevisiae* increased TAC levels. On the other hand, it decreased MDA, PCO, and DPC, which had risen due to Cd exposure. Furthermore, it increased the expression of *proPO*, which was reduced by Cd exposure, and decreased the expression of *LSZ* and *MT*, acting in the opposite direction of Cd exposure alone. These findings demonstrated that feeding *S. cerevisiae* effectively reduces the Cd from crayfish and could be used to develop Cd-free crayfish-based foods.

## Introduction

Over the past few years, heavy metal-caused pollution in aquatic systems has drawn increasing attention^1^. Cadmium (Cd) is a non-decomposable pollutant that has brought concerns about environmental issues and its impact on human health worldwide^2,3^. Cd's toxicity can be bio-amplified in the food chain^4^. Cd can trigger several harmful changes, including biochemical changes, morphological disruptions in cellular structures, and physio-biological dysfunctions^5,6^ through stimulating increased accumulation of reactive oxygen species (ROS) in living organisms^7–9^.

Oxidative stress from excess reactive oxygen species suggested that DNA damage and protein oxidation may be responsible for cadmium toxicity^10–13^. Excessive ROS accumulation in the antioxidant defence system was brought on by an imbalance between ROS production and removal^14–16^. In addition to impairing cellular processes, ROS also mediates the signaling pathways involved in physiological processes^17–19^. The prophenoloxidase (proPO) activating system (proPO-AS) is a special invertebrate defence mechanism that is essential for melanization in response to environmental stress^20^. Based on Söderhäll and Smith^21^ reports, the inactive proPO zymogen in granular and semi-granular hemocytes is initially transformed into active phenoloxidase (PO) before catalyzing the oxidation of phenols to quinines, which are later polymerized to melanin^22^. Hemocytes are the main structural source of proPO in crustaceans, according to Cerenius and Söderhäll^23^, Terwilliger and Ryan^24^, Matozzo and Marin^25^. Furthermore, Cd-induced damage to lysosomal membranes leads to the release of lysozyme (LSZ) based in granular hemocytes^26^. Additionally, lysozyme is employed as a sensitive and trustworthy biomarker to assess the toxicity of environmental contaminants^27^. Additionally, the main role of the metal ion detoxification processes is played by the protein metallothionein (MT), a low-molecular-weight, cysteine-rich, and metal-binding protein^28,29^. Importantly, it has been shown that metals play a major role in regulating *MT* mRNA expression at the transcription level^28,30^ and that inducing MT is thought to be essential for reducing Cd toxicity, such as oxidative damage^31^.

Red swamp crayfish (*Procambarus clarkia*) is a commercially significant freshwater cultured species^32^. The main reason for regarding this species as a typical bioindicator of toxic pollutants in aquatic environments is that they have a lengthy lifecycle, widespread distribution, and fairly simple anatomy^33,34^. According to an earlier study, the hepatopancreas are vital, multifunction organs for crustaceans' nutrient absorption, metabolism, and immune function^35^. The hepatopancreas was introduced as the core target of numerous environmental stresses^36,37^.

It has been reported that environmental Cd accumulates in crayfish tissues. Pollution levels in the environment have shown a positive correlation with concentrations in tissue samples^38^. Tissue levels were frequently found to be positively correlated with distance from the source of pollution^39–41^. The Cd is taken up and accumulated by crayfish from both the environment and food^42^. The Cd accumulation and detoxification are primarily carried out by the hepatopancreas in crayfish^43^ and other crustaceans^44,45^. The highest accumulation of Cd in crustaceans exposed to various Cd concentrations has been reported in the gills^46^.

To determine the contamination of these metal contaminants, the hepatopancreas and bioaccumulation ratio between the hepatopancreas and abdominal muscle of *P. clarkii* exposed to heavy metals have been used^47–49^. Bioremediation is an effective, broad, cost-effective, and environmentally acceptable cleanup technology^50^. Over the past several decades, there has been extensive research on cadmium removal by biosorption^51,52^. Microorganisms like fungi, algae, or bacteria can remove cadmium during biosorption^53,54^. Heavy metal (e.g., Cd^2+^, Pb^2+^, Cu^2+^) elimination by *Saccharomyces cerevisiae* has been attributed to their adsorption capacity. *S. cerevisiae* has previously been used to remove lead from milk, and research has shown that it is a natural biosorbent that can do so^55^. The CAD1 protein of *S. cerevisiae* contains domains bZIP and PAP1 that are closely related to Cd overexpression resistance^56^. *S. cerevisiae* has a part in heavy metal detoxification mechanisms, including sulfur refactoring pathways in yeast, glutathione production mechanisms, and reducing oxidative stress-related gene and protein expression^57^. Heavy metal ions with decreased toxicity of metallothionein and plant chelating peptide binding were later discovered^58^. Previous research has demonstrated that high heavy metal contamination in wheat can be overcome by using *S. cerevisiae* as a native remedy^59^. Amirnia et al.^60^ demonstrated the efficacy of removing heavy metals from industrial effluents using living yeast cells in a self-contained continuous adsorption system.

Furthermore, the use of *S. cerevisiae* to remove cadmium from fish such as Cyprinidae has a highly promising future^61–63^. At present, there are no reports on the possible role of *S. cerevisiae* in the attenuation of heavy metal concentrations in crayfish. On the other hand, several investigations in various aquaculture species have been undertaken, and the potential of *S. cerevisiae* as a probiotic has been investigated^64–66^. Similarly, Siddik, et al.^64^ shown in juvenile barramundi, *Lates calcarifer*, that a basal meal supplemented with 1% *S. cerevisiae* and 1% *Lactobacillus casei* alters physiological performance and promotes gut microbiome. The main objective of this study is to examine the ability of *S. cerevisiae* as a bioremediation method to combat Cadmium stress. The effects of a basal diet with free and varied dosages of *S. cerevisiae*, as well as different feeding days, on crayfish cadmium removal were examined across a 10-day exposure trial and a 12-day cadmium removal experiment. To validate our removal test, we also measured total antioxidant capacity (TAC) and oxidative stress indicators such as malondialdehyde (MDA), protein carbonyl derivates (PCO), and DNA–protein crosslink (DPC). In addition, four additional conformations were studied, as well as the mRNA expression levels of lysozyme (*LSZ*), metallothionein (*MT*), and prophenoloxidase (*proPO*) in crayfish. The research findings are expected to open new avenues for developing feed additives, heavy metal removal technology, and new removal agents for crustaceans and aquatic animals.

## Materials and methods

### Crayfish sampling

In September 2020, 480 adult male crayfish with similar sizes (10.011.10 cm length; 20.300.31 g wet weight) were obtained from a crayfish company (Yibin Haide Fishery Technology Co., LTD) in Yibin, China.

### Yeast strains and growth conditions

*S. cerevisiae* was isolated from the intestinal tract of Procambarus Krai and was preserved in the Department of Agriculture, Forestry and Food Engineering, Yibin University, Reserve number (FYBNL) M2020121. *S. cerevisiae* was grown in yeast peptone dextrose adenine (YPDA; Qingdao Biological Technology Co., Ltd., Qingdao, China) overnight at 30°C before being resuspended in sterile phosphate-buffered saline (PBS). The plate dilution counting method was used to determine the colony forming units (CFU) per milliliter of *S. cerevisiae* culture. A mixture of 1% and 5% *S. cerevisiae* powder was made by grinding basic feed into powder and mixing it with *S. cerevisiae* powder. A specified amount of water was added, mixed, and heated on the electric stove for several minutes^67,68^. The feed was vacuum-dried overnight at 30 °C and stored at 4 °C. Every 3 days, the feed was prepared in the same way.

### Feeding frequency and cadmium exposure

The experimental crayfish were divided into six boxes, each holding 80 crayfish, after seven days of acclimatization. The boxes were allocated into three groups at random: two treatment groups (TGs) and one control group (CG). For 28 days, the treatment group was fed *S. cerevisiae* feed (1% and 5%), whereas the control group was fed the basal commercial feed^68^. To clearly examine the removal effect of *S. cerevisiae* on crayfish, sampling analysis was performed on the first, fourth, eighth, twelfth, and twenty-first days of acculturation.

For 10 days, all crayfish were kept in plastic aquaria (90 cm60 cm25 cm) of aerated tap water containing Cd (1.450 mg L^−1^) and 12 days without Cd. This experiment was performed at Yibin University in the Chinese province of Sichuan. The Cd concentrations were 20 times higher than the national standard for fishery water quality. Crayfish were fed a base diet for the first ten days and then switched to a different diet for the next twelve days as shown in Table 1.

**Table 1:** The basic of crayfish diet composition.

Items	Feed composition	Percent (%)
Feed the basal diet for the first 10 days	Fish meal	37
	Soybean meal	28
	Wheat flour	15
	Corn flour	10
	Fish oil	2.5
	Soybean oil	2.5
	multivitamin and mineral elements	3
	Sodium carboxymethylcellulose	2
Feed different diets for the next 12 days	Basal diet plus 1% *S. cerevisiae*	
	Basal diet plus 5% *S. cerevisiae*	

The nutritional levels of basal diet were 28.5% crude protein, 6.2% crude fat, 41.9% carbohydrate and 8.1% crude ash.

Basic feed without *S. cerevisae* (Group 1), *S. cerevisae* at 1%, and 5% supplemented in basic feed (Groups 2 and 3) were the three food groups. (2nd and 3rd groups). Each group had three replicates, each with 80 animals. Six parallel samples from each group were collected every four days for a total of four times in the removal experiment.

### Graphite furnace atomic absorption spectroscopy (GF-AAS)

Cd measurements in crayfish tissue were performed using a Perkin-Elmer Analyst 800 Atomic Absorption Spectrometer outfitted with a Zeeman background correction device and an electrothermal atomizer transversely heated graphite tube (THGA)^69^. Electrodeless discarge lamps (EDLs, Perkin-Elmer) at 282.3 nm (slit width 0.7 nm) were used as radiation sources for the Cd. At room temperature, twenty-microliter aliquots of the material were injected into a graphite tube, followed by a two-step drying, pyrolysis, and atomization (one step each). The graphite tube was finally cleaned. Dilution of a certified 1000 mg L^−1^ Cd monoelement standard solution (Trace Cert Fluka) was used to generate standard solutions for calibration curves. Reagent blank was used to dilute all solutions. The method's detection limit, as determined by the Cd calibration curve, was Cd, 0.19 mg kg^−1^.

### Total antioxidant capacity (TAC) determination

The TAC was measured using the TAC Assay Kit according to the manufacturer's instructions. A Fluko Superfine Homogenizer at 1000 rpm for about 30 s was used to homogenize 300 µl of each sample with 106 hemocytes/mL. The samples were centrifuged at 12,000 g for 4 min at 4 °C. Then, working solutions containing 20 l of catalase and 170 l of 2,′-Azinobis-(3-ethylbenzthiazo-line-6-sulfonate) (ABTS) were added to 10 l sample solutions and kept at room temperature for 10 min. A microplate reader was used to measure the TAC at 414 nm (Spectramax M5 multimode microplate reader, San Francisco, CA, USA). We used soluble Trolox as a reference. The corresponding Trolox/mg protein concentration in individual samples was used to calculate the results^70^.

### MDA, DPC, and PCO assay

The thiobarbituric reactive species (TBARS) assay was used to measure the production level of MDA^71^. PCO and DPC measurements were performed following standard protocols introduced by Li et al.^72^. PCO contents were quantified using 2,4-dinitrophenylhydrazine (DNPH), which reacted with protein carbonyl derivates to form 2,4-dinitrophenylhydrazone. OD values were computed at 370 nm, and their expression (nmols of carbonyl groups/mg protein) was based on a molar extinction coefficient of 22,000 M/cm for aliphatic hydrazones. The concentration of DPC was determined using KCl-SDS, which was used to precipitate the crosslink and separate free DNA from protein-bound DNA. To combine with DNA, Hoechst 33,258 was added. The fluorescence was measured at a specific wavelength (excitation: 350 nm; emission: 460 nm). The fluorescence ratio was calculated as a percentage of protein bound to total DNA.

### Total RNA extraction and cDNA synthesis

Total RNA was extracted away from hemocytes originating from animals at different treatments thanks to the Trizol Lysis Reagent (TaKaRa, Dalian, China) following the manufacturer's protocol. Then, the concentration and purity of Total RNA extracts were estimated using a BioSpectrometer fluorescence (Eppendorf, Hamburg, Germany) and 1.2% agarose gel electrophoresis, and genomic DNA was removed by DNase I (TaKaRa, Dalian, China) digestion. First-strand cDNA was synthesized from 2 µg of total RNA using a cDNA synthesis kit (TaKaRa, Dalian, China).

### Real-time quantitative RT-PCR of proPO, LSZ, and MT

The designation procedure for quantitative fluorescent RT-PCR primers was as per the transcriptome sequences using Primer 5 software (Table 2).

**Table 2:** *proPO*, *LSZ*, *MT,* and *β-actin* primers used in Real-time PCR.

Genes	Sequences of specific primers (5′–3′)	Length (nt)	TM (°C)
Prophenoloxidase (*proPO*)	**F-** TGCCTTAGGGGTGTTTTA	18	61
	**R-** CAGGGTGACTGGTCTTGG	18	
lysozyme (*LSZ*)	**F-** GAGGATGTGGTCGTGGGTGA	20	67
	**R-** ATTGGTCGTTCTAATGCCGC	20	
metallothionein (*MT*)	**F-** CTGCTCTGGAGTAGGGTTCG	20	68
	**R-** GCTCTCGTGAAGACTTATGGCG	22	
*β-actin*	**F-** GCTGTTATGGTTGGTATGGGTC	22	67
	**R-** TCGGTGAGAAGAACGGGG	18	

In order to compare the relative levels of expression of *proPO, LSZ,* and *MT* in the samples, the housekeeping gene *β-actin* was also amplified with the same cDNA samples. The RT-qPCR was carried out in a total volume of 20 µl, containing 10 µl of 2 × SYBR Premix (TaKaRa), 0.4 µl of each primer (10 µM), 0.4 µl ROX dye П, 2 µl of the diluted cDNA and 6.8 µl ddH_2_O. The thermal profile for RT-qPCR was 30 s at 95 °C for 1 cycle, 5 s at 95 °C, 30 s at 60 °C, and 30 s at 72 °C for 40 cycles. An ABI 7500 real-time detection system (Applied Biosystems, Foster City, CA, USA) was utilized to run the RT-qPCR using SYBR Premix Ex Taq II (Takara, Dalian, China) based on the manufacturer's protocol. The proposed experiments were done in triplicates involving NTC. Fold change for the gene expression relative to controls was determined by the 2^−ΔΔCt^ method^73^.

### Statistical analysis

The data were obtained for statistical analysis by analysis of the variance (ANOVA) to compare the statistical differences between the experimental groups at *p* < 0.05 statistical significance level using SPSS software.

### Ethics statement

The animal study was reviewed and approved by the Administration Committee of Experimental Animals, Sichuan Province, China, and the Institutional Animal Care guidelines of Yibin University, China.

## Results

### Cd concentration in crayfish

The Cd concentration in the edible parts of the crayfish feeding basal diet was 14.67–14.82 mg/kg, as shown in Table 3, and there was no significant declining trend throughout the process of 0–21 d water purification (basal feed).

**Table 3:** Effect of *S. cerevisiae* feeding on Cd concentration in the edible parts of crayfish.

Group	Percent *S. cerevisiae* in additive	The concentration of Cd in crayfish during the removal period (mg/kg)				
		0d	4d	8d	12d	21d
1	0%	14.77 ± 0.32^Aa^	14.67 ± 0.32^Aa^	14.73 ± 0.18^Aa^	14.82 ± 0.23^Aa^	15.15 ± 0.46^Aa^
2	1%	14.59 ± 0.33^Aa^	13.30 ± 0.25^Bb^	13.32 ± 0.65^Bb^	12.44 ± 0.31^Cb^	8.29 ± 0.73^Cb^
3	5%	15.08 ± 0.62^Aa^	13.46 ± 0.65^Bb^	12.23 ± 1.21^Cc^	10.60 ± 0.54^Dc^	5.06 ± 0.87^Ed^

In the same row, values with different capital letters superscripts mean significant differences (P < 0.05). Values with different lowercase letter superscripts in the same column indicate significant differences (P < 0.05).

Simultaneously, *S. cerevisiae* at 1% supplemented in basal diet feeding crayfish had a removal effect, and there had fallen by 43.1% after 21 days of water purification (1% of *S. cerevisiae*). The clearance rates on the fourth and eighth days were 8.84% and 8.7%, respectively, with no significant difference between them but a significant change when fed 1% *S. cerevisiae* on day 0. The best clearance rate, however, was 66.45% when fed 5% *S. cerevisiae* on the 21st day. The elimination rates were significantly different at 0, 4, 8, 12, and 21 days. The Cd level of the crayfish in the control group was significantly higher than that of the crayfish fed 1% and 5% S. cerevisiae, as indicated in Table 3. Despite this, no significant differences were observed among them after four days of feeding in water. On the 8th, 12th, and 21st days, the Cd concentration of crayfish with varied proportions of *S. cerevisiae* was significantly lower than the control group, and the Cd concentration of *S. cerevisiae* with different ratios was also statistically different. Finally, after 21 days, the 1% and 5% *S. cerevisiae* showed the most significant activity in eliminating Cd, with Cd concentrations of 8.29 and 5.06 mg/kg, respectively.

### The TAC level of the hemocytes of crayfish

**Figure 1:**
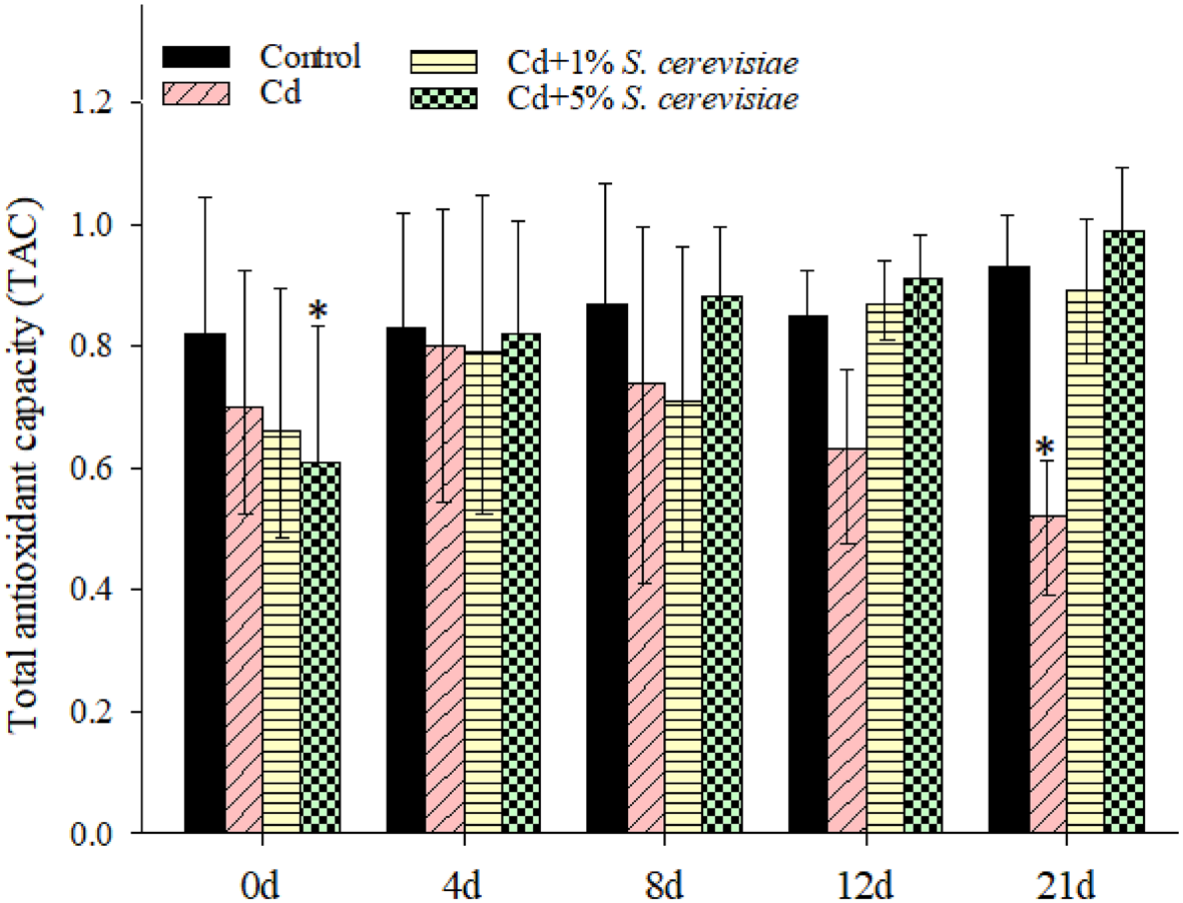
Total antioxidant capacity (TAC) in crayfish hemocytes at different treatments. Data are means ± SD, n = 6 crayfish per treatment at each time point. Compared to the control group, significances are indicated by *p < 0.05 and **p < 0.01.

Except for the group of Cd + 5% *S. cerevisiae* on 0 d and Cd in 21 days, no significant changes in TAC levels were found following Cd exposure, Cd + 1% *S. cerevisiae*, and Cd + 5% *S. cerevisiae* throughout the treatment periods, as shown in Fig. 1. TAC levels for Cd, on the other hand, decreased significantly, falling by 63% and 52%, respectively, compared to controls. The TAC steadily increased with *S. cerevisiae* concentrations, peaking at Cd + 5% *S. cerevisiae*. TAC in crayfish hemocytes was reduced dose-dependently by Cd.

### The MDA, PCO, and DPC levels in hemocytes of crayfish

**Figure 2:**
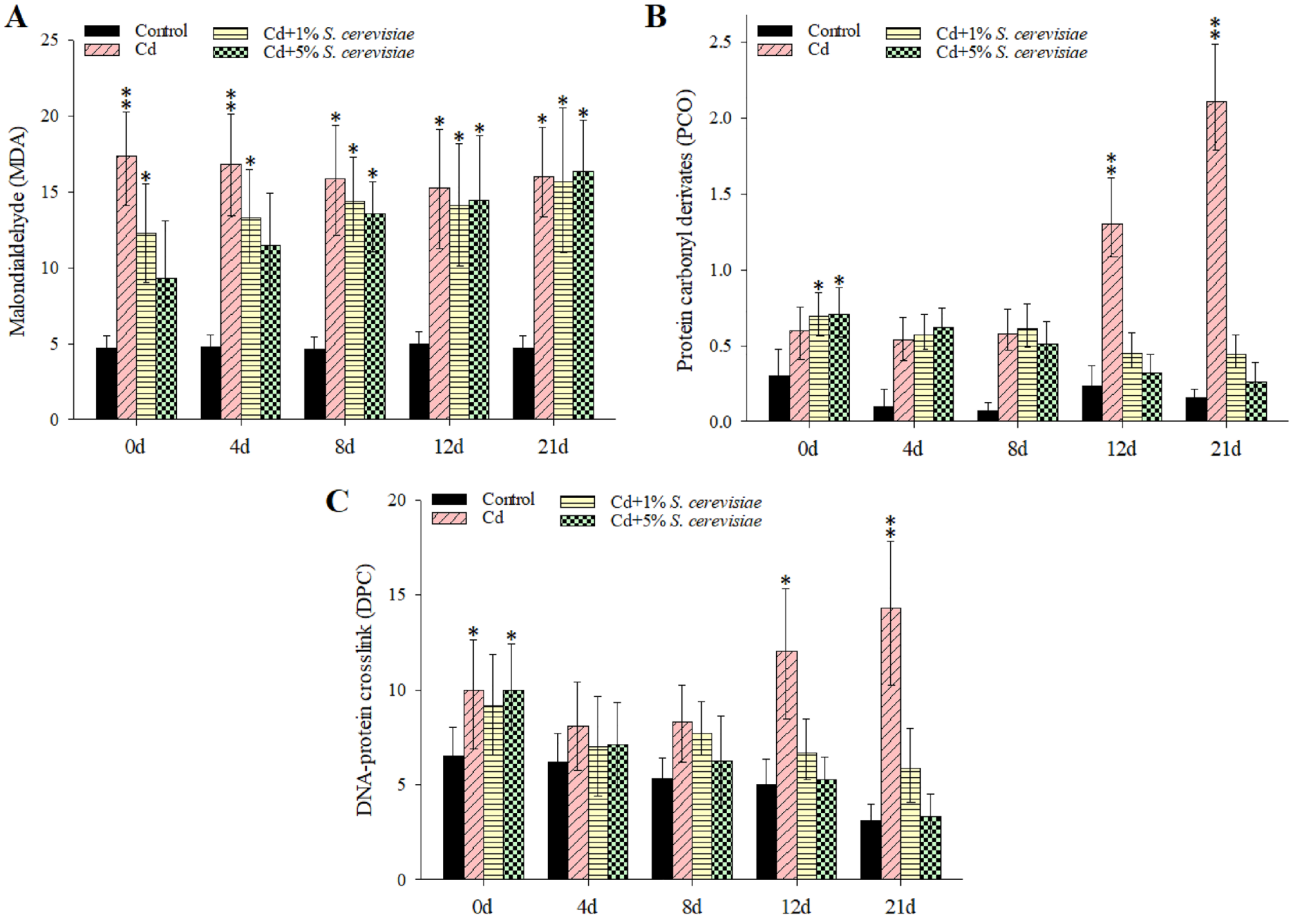
Effect of Cd and *S. cerevisiae* addition on (**A**) MDA, (**B**) PCO contents, and (**C**) DPC coefficient in crayfish hemocytes. Data are means ± SD, n = 6 crayfish per treatment at each time point. Compared to the control group, significances are indicated by *p < 0.05 and **p < 0.01.

The MDA content of hemocytes increased significantly (p 0.05) when compared to the control group (Fig. 2A). MDA levels in crayfish exposed to Cd increased with time and were 4.71 and 16-fold higher than in controls, respectively. MDA decreased with 1 and 5% *S. cerevisiae* additions compared to Cd treatment free any *S. cerevisiae*, but it remained higher than the control. As observed in Fig. 2B, PCO levels in hemocytes increased dramatically over time as Cd concentration increased. After Cd (1.450 mg L^−1^) exposure, the PCO level increased to 2.11-fold higher than the controls, especially on the 21st day. With time increasing, the 1 and 5% *S. cerevisiae* additions not showed any decreases in PCO until eight days, but the PCO level decreased over an extended period ( 12 and 21 days) which was more remarkably with 5% *S. cerevisiae* addition.

The DPC level was considerably increased after Cd exposure compared to the control groups (Fig. 2C). DPC levels increased with time and exposure to greater Cd concentrations when compared to the control. With 1 and 5% *S. cerevisiae* additions and increasing time, the DPC level was significantly decreased, more notably with 5% *S. cerevisiae* additions.

### The expression status of *proPO* in hemocytes of crayfish

**Figure 3:**
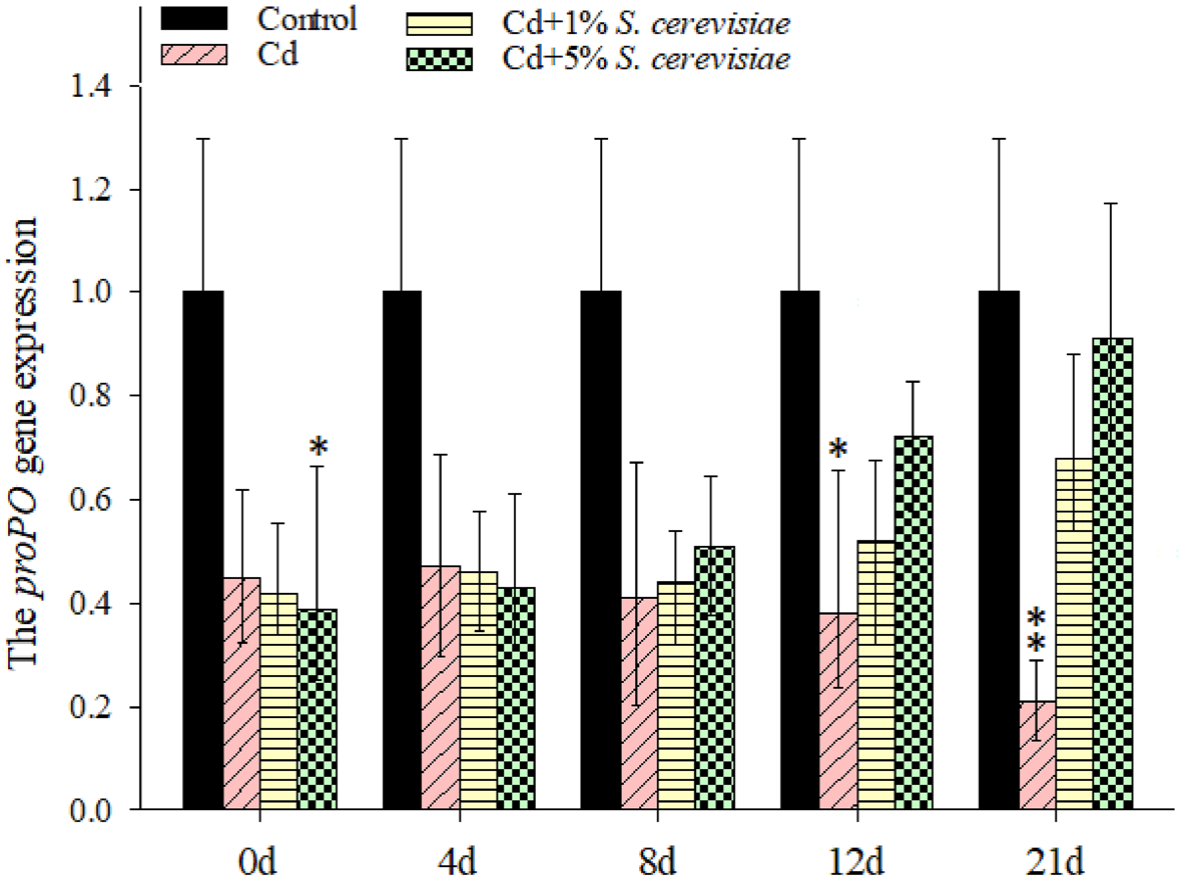
The expression levels of *proPO* in the hemocytes of crayfish. Data are means ± SD, n = 6 crayfish per treatment at each time point. Compared to the control group, significances are indicated by *p < 0.05 and **p < 0.01.

Along with the increase in Cd content, there was a general downward trend in *proPO* expression levels (Fig. 3). When compared to the control group, proPO expression levels were significantly reduced, especially after 21 days (p 0.05). The expression of *proPO* in crayfish hemocytes containing Cd has decreased dramatically over time when compared to those that do not contain Cd. With the 1 and 5% *S. cerevisiae* additions, the *proPO* were significantly up-regulated beginning from the eighth day and with extended time compared to Cd without any of *S. cerevisiae* additions. The increase in *proPO* was remarkably in 5% more than 1% *S. cerevisiae* addition. Cd exposure inhibited the expression level of *proPO* in crayfish hemocytes.

### The expression levels of *LSZ* and *MT* in hemocytes of crayfish

**Figure 4:**
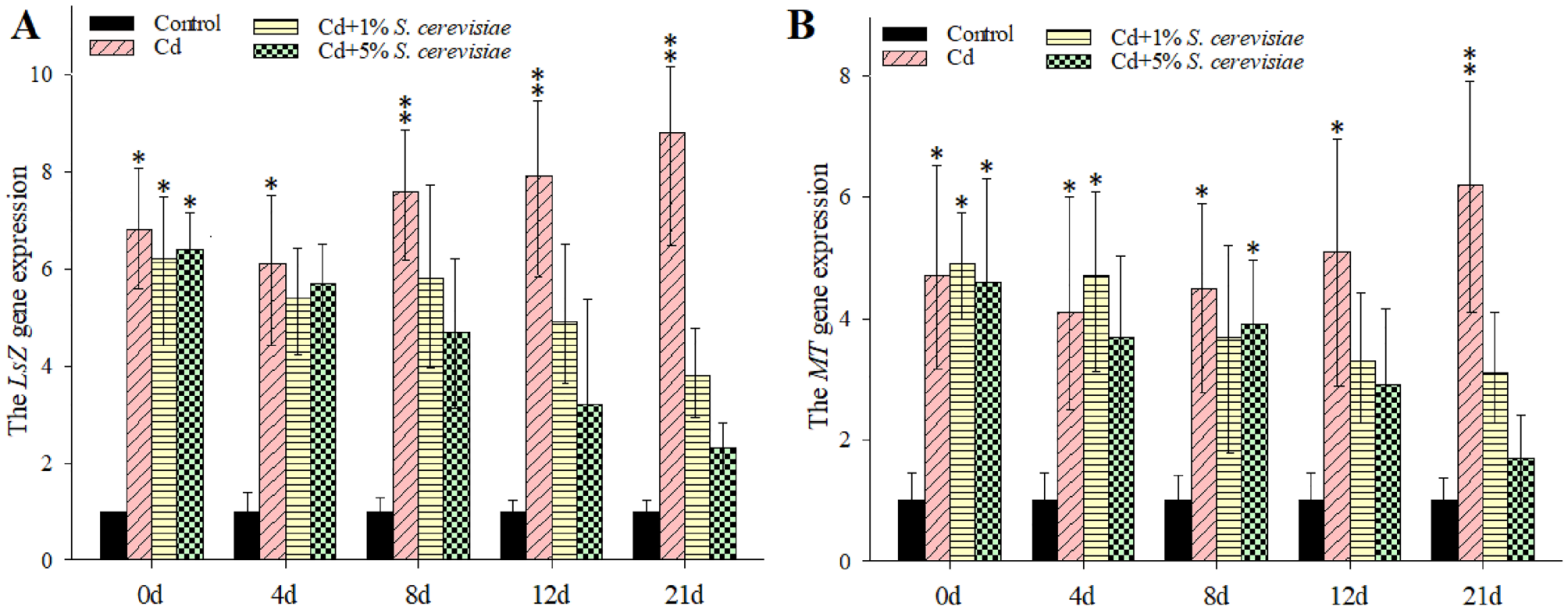
The expression levels of *LSZ* and *MT* in crayfish hemocytes. Data are means ± SD, n = 6 crayfish per treatment at each time point. Compared to the control group, significances are indicated by *p < 0.05 and **p < 0.01.

*LSZ* mRNA expression level demonstrated a dose-dependent response to Cd treatment (Fig. 4A). *LSZ* expression level increased rapidly beginning at 8 d in Cd treatment groups, which recorded a high level in 21d with 8.8-fold compared to control. Both 1 and 5% *S. cerevisiae* additions led to a decrease in the expression of *LSZ*, which was more in 5% than 1% *S. cerevisiae* addition. Cd treatment significantly increased *LSZ* mRNA expression levels, while *S. cerevisiae* additions decreased it in the hemocytes in a dose-oriented manner (Fig. 4A). Cd treatment resulted in a significant increase in *MT* mRNA expression levels in the hemocytes in a dose-oriented manner with a maximal response at 21 d at the exposure range of 6.2-fold compared to the control (Fig. 4B). Both 1 and 5% *S. cerevisiae* additions led to a decrease in the *MT*, especially at 12th and 21st d. Cd exposure significantly incremented *MT* mRNA expression levels, whereas *S. cerevisiae* additions did not decrease this expression in the hemocytes in a dose-oriented manner.

## Discussion

Cadmium has a strong bioenrichment effect in crayfish, and its biometabolic half-life is long. The majority of heavy metals are extremely toxic and non-biodegradable. Cu(II), Cd(II), Zn(II), and Pb(II) maximum permissible concentrations in China are 2.0, 0.1, 5.0, and 1.0 mg/L, respectively^74,75^. As a result, in order to meet increasingly stringent environmental quality standards, they must be removed from waste effluents. To treat such effluents, various methods such as chemical precipitation, ion exchange, electrodeposition, membrane separation, and adsorption have been used^76^. Traditional chemical precipitation was the most cost-effective method, but it is inefficient. Ion exchange and reverse osmosis were both effective in general but had high maintenance and operational costs^77^. Adsorption, particularly with low-cost natural sorbents, is also one of the few promising methods for removing toxic metals from aqueous environments. The previous research of Zhang et al.^78^ demonstrated the efficacy of Cadmium removal using MgCl_2_-modified biochar (MgC600) derived from crayfish shell waste.

Bioremediation is a natural alternative process to incineration, catalytic degradation, adsorbent use, physical removal, and ultimately pollutant destruction^79,80^. Microorganisms are a biological tool for metal removal because they can be used to remove, concentrate, and extract heavy metals from polluted aquatic habitats^81^. The bioremediation strategy is based on biological agents' high metal-binding ability, which aids in the extraction of heavy metals from very dilute solutions. Bioremediation using microorganisms is very beneficial and can also adapt to extreme conditions in polluted areas. Because microorganisms act on pollutants even in very dilute solutions, bioremediation with microorganisms is very beneficial and can also adapt to extreme conditions^79^.

Our study found that Cd could not be eliminated quickly when crayfish were raised in pure water without *S. cerevisiae*, which largely agrees with the previous studies' results^82,83^. The concentration of Cd in crayfish with temporary feeding or conventional diet could not be effectively removed by natural metabolism, which is also applied to oysters^84^. There is a general agreement over the benefits of *S. cerevisiae* in the biosorption of limited concentrations of Cd and alleviation of contaminated foods with the employment of green technologies under the disguise of a natural, low-cost, and abundant sorbent^85^. The mechanism of Cd toxicity in *S. cerevisiae* has been studied^86^ in conjunction with Cd-induced UPR, intracellular ROS levels, and cell death, all of which may play important roles in Cd-induced toxicity, though the mechanism by which *S. cerevisiae* removes cadmium from crayfish is not well studied. To the best of our knowledge, the distribution of Cd in crayfish is controlled by the p38 Mitogen-activated Protein Kinase (MAPK) by modulating the accumulation of Cd in different crayfish tissues under Cd-stressed conditions^87^. We hypothesized that *S. cerevisiae* and crayfish cells might have competitive adsorption for cadmium. The *S. cerevisiae* species was used to remove Cd from milk obtained at the highest Cd removal (70%) rate. This proportion was at 80 μg/L of Cd concentration in milk samples after the final storage time of the four-day^88^. As shown in Table 1, when *S. cerevisiae* was used in crayfish feed, the highest cadmium removal rate was only 29.7% at 5% of *S. cerevisiae* but higher than 1%. Therefore, it is assumed that the increased concentrations of *S.cerevisiae* biomass provided a more binding site for Cd and hence a higher capacity for Cd removal. Cd, as is well known, cannot produce free radicals directly. On the other hand, Cd can indirectly stimulate the production of ROS through the superoxide radical and the hydroxyl radical^89^. Furthermore, an intriguing mechanism was presented to explain Cd's indirect role in ROS generation, in which Cd was thought to replace Fenton-active metals such as iron and copper in cytoplasmic and membrane proteins (e.g., Ferritin), increasing the number of freely iron and copper ions that participate in oxidative stress via Fenton reactions^90^.

Importantly, Wätjen and Beyersmann^91^ back up the preceding findings. Excess ROS are normally eliminated by the antioxidant system to maintain the body's redox status^92^. However, when the generation of ROS surpasses the body's antioxidant defences, lipid peroxidation, protein modification, DNA damage, and other oxidative effects are induced^93^. Membrane lipid peroxidation has been identified as one of the functional repercussions of oxidative damage^94,95^. MDA, a traditional marker of lipid peroxidation, reflects the degree of oxidative damage^96^. As a result, the severity of oxidative stress caused by Cd can be determined based on changes in MDA levels in organs or cells^97,98^.

Numerous studies have shown that Cd-induced reduction of antioxidant enzyme activities can inhibit the scavenging process of ROS, which can lead to an increase in MDA levels in cells or organisms^93,99,100^. An earlier study discovered that Cd could induce ROS generation in the hemocytes of the crab *S. henanense*^26^. In the present study, Cd exposure increased the level of MDA compared to Cd exposure with *S. cerevisiae* as feed additives, indicating the efficiency of *S. cerevisiae* in removing the Cd effect from crayfish cells. As we discovered in this study, Cd (21 d) significantly inhibited TAC activity in crayfish hemocytes, whereas TAC activity was recovered by using *S. cerevisiae* as feed additives. Previous research by Zhou et al.^28^ demonstrated that Cd (2.900 mg L1, 14 and 21 days) exposure significantly inhibited TAC activity by increasing MDA concentrations in crab hemocytes.

Also, the same previous study by Zhou et al.^28^ found a decrease in TAC activity accompanied by an increase in MDA levels, which is consistent with the above reports. Furthermore, there is evidence that Cd exposure can cause antioxidant enzyme inhibition, GSH depletion^101^, and the potential for genotoxic and cytotoxic effects owing to an increase in the ROS^102^. Carbonyl is a typical biomarker for protein damage following exposure to the ROS, which negatively affects the amino acids in the protein side chains for producing carbonyl in the protein^72^. DNA–protein crosslinks (DPC) are thought to be constantly formed in cells during metabolism, such as via the interaction of glucose-6-phosphate with lysine amino groups, and ROS also produces a high percentage of DPC^103^. In our study, Cd exposure resulted in significant increases in DPC and PCO levels, while using *S. cerevisiae* as feed additives resulted in a decrease, indicating their efficiency in removing Cd toxicity in crayfish cells.

Prior research by Zhou et al.^28^ revealed that the effects of Cd on the accumulation of the PCO and the emergence of DPC in crab hemocytes were quite obvious. Previous research found that acute Cd (58 and 116 mg L1, 7 d) exposure could cause a significant increase in PCO and DPC in *S.henanense* sperms^97^. Thus, it was reported that Cd could induce DPC generation through (1) excessive ROS-induced oxidative damage to amino acids and proteins and (2) Cd direct interference with the covalent combination of amino acids in the protein and the nucleotide of DNA^72^. Evidence has proven that Cd exposure engenders cell necrosis, characterized by cell membrane disintegration followed by intracellular content dissemination^104,105^.

ROS generation is an emerging step in Cd-induced cytotoxicity which is followed by a decrease in mitochondrial membrane potential^106^. Mitochondrial damage is usually accompanied by the activation of caspases and programmed cell death^107,108^. Overall, it has been demonstrated that excessive ROS production is associated with lipid, protein, and DNA damage, resulting in impaired cellular structure and functions. According to Qin et al.^109^, *S. henanense* hemocytes are classified into large granular, semi-granular, and hyaline. Acute Cd also affected hemocyte organelles. Cd causes oxidative stress in aquatic organisms^102^ and suppresses immune responses^110^. In invertebrates, particularly crustaceans, the prophenoloxidase activating system is critical for immunity^111^. A serine protease cascade response would convert *proPO* to active *PO*, similar to the complement activating system in vertebrates^112^. Ammonia was found to have a significant impact on *proPO* gene expression^113^. The activity of *proPO* was significantly reduced in freshwater crayfish *Procambarus clarkii* after exposure to copper^103^. Furthermore, Cd exposure reduced *proPO* gene expression in crayfish hemocytes in the current study. The application of *S. cerevisiae* as feed additives has intensified the expression level of *proPO*. This phenomenon was consistent with the Sun et al.^114^ report, which revealed that Cd exposure downregulated the expression of *proPO* in the hepatopancreas of the crab *S.henanense*.

Aquatic defence effectors such as LSZ and MT can respond well to stress factors by protecting organisms from serious harm^115^. LSZ is an essential lysosomal enzyme capable of lysing bacterial membranes, avoiding the risk of bacterial infections^116^. According to Tyagi et al.^117^, *LSZ* expression levels were increased in the black tiger shrimp Penaeus monodon after the bacterial pathogen challenge. Furthermore, when the clam *Mactra veneriformis* was exposed to Cd and Hg for 5 and 7 days, *LSZ* expression levels increased^115^. On the other hand, in the study of Jakiul Islam et al.^118^ on the European seabass, *Dicentrarchus labrax*, they found an increase in *LSZ* expression levels during extreme cold events at various salinities. Here, we discovered that *LSZ* expression was up-regulated in crayfish hemocytes under Cd stress. *LSZ* expression levels increased rapidly beginning at 8 days in Cd treatment groups, reaching a peak in 21 days with an 8.8-fold increase compared to controls. Both 1 and 5% *S. cerevisiae* additions resulted in a decrease in *LSZ* expression, with the 5% *S. cerevisiae* addition being more effective than the 1% *S. cerevisiae* addition. In contrast, it was down-regulated when the feed additive *S. cerevisiae* was included in the diet. This finding suggests that *S. cerevisiae* effectively mitigates the negative effects of Cd exposure. *LSZ* expression levels were up-regulated in the hemocytes of crab *S. henanense* under Cd stress (1.450, and 2.900 mg L ^−**1**^ ) in a previous study by Zhou et al.^28^. However, previous research found no significant changes in LSZ activity when crabs were subjected to Cd stress in *S. henanense* hemocytes^26^.

MT is a high-affinity ligand for Cd absorption, transport, and detoxification^119^. It has a high antioxidant capacity and protects cells from the cytotoxic effects of ROS^120^. Earlier studies have shown that Cd-mediated *MT* mRNA expression scavenges ROS production under Cd stress^121, 122^. Furthermore, there is evidence that *MT* expression levels increased significantly after 3 days of Cd challenge in the clam *Mactra veneriformis*^115^. Exposure to Cd has been associated with increased *MT* expression in Pacific oysters *Crassostrea gigas*^123^, hard clams *Meretrix lusoria* (Chang et al., 2007), and scallops *Argopecten irradians*^124^. In this experiment, we discovered that *MT* mRNA expression levels were induced, and more transcription of *MT* mRNA was activated in crayfish treated with high Cd concentrations. In contrast, it was downregulated with Cd when *S. cerevisiae* was used as a feed additive. Zhou et al.^28^ observed that high Cd concentrations induced *MT* mRNA expression levels and activated more *MT* mRNA transcription in *S. henanense* than in low Cd concentrations. Fang, et al.^115^ reported a similar result in the clam *M. veneriformis*. It is hypothesized that this is MT's defence mechanism against Cd toxicity. Thus, we identified the importance of *S. cerevisiae* additive in regulating *MT* expression and providing adequate protection from Cd toxicity in the examined crayfish.

## Conclusion

**Figure 5:**
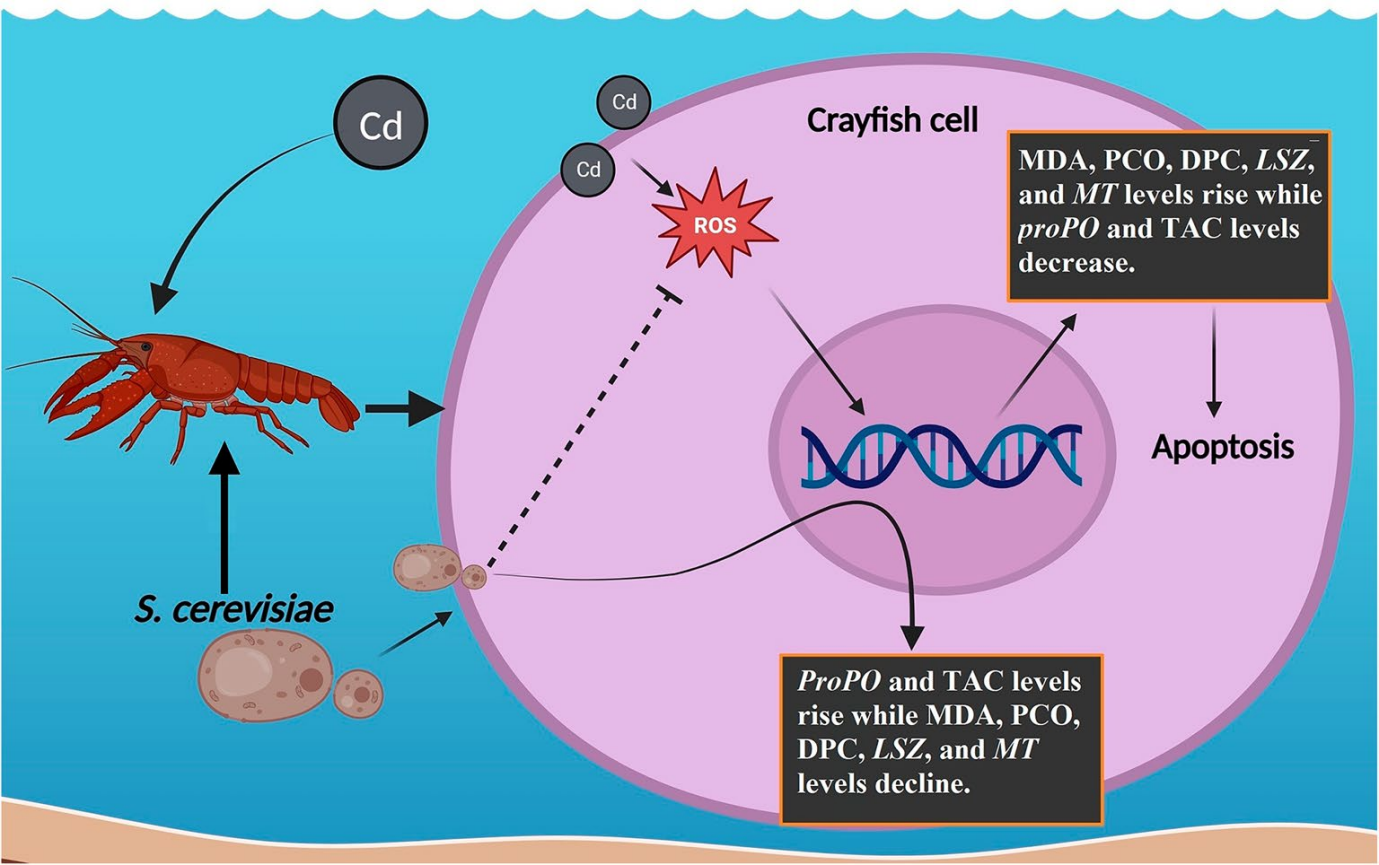
The effect of *S. cerevisiae* additions on the differential expression of oxidative-related genes in *P. clarkii* in the presence of Cd toxicity

Despite the fact that the third biggest known freshwater crayfish species has been shown to have high levels of harmful metals, our understanding of the potential dangers of consuming this species lags well behind that of finfish. Because China produces the most crayfish in the world, safe solutions to counteract the hazards of ongoing heavy metal stressors as heavy metals build must be improved. The goal of this study was to use *S. cerevisiae* as a bioremediation agent to mitigate the negative effects of Cd on crayfish (*P. clarkia*). The results showed that *S. cerevisiae* at 5% supplemented in fundamental feed had the best removal effect, with Cd removal rates at days 4, 8, 12, and 21 being 12%, 19%, 29.7%, and 66.45%, which were significantly higher than the crayfish's basal diet. TAC levels were increased by the addition of *S. cerevisiae*. On the other hand, it reduced MDA, PCO, and DPC levels, which had risen as a result of Cd exposure. Furthermore, it increased *proPO* expression, which was decreased by Cd exposure, and decreased *LSZ* and *MT* expression, acting in the opposite direction as Cd exposure alone as shown in Fig. 5. These findings show that feeding *S. cerevisiae* effectively reduces crayfish Cd levels and could be used to develop Cd-free crayfish-based foods.

## Data Availability

The datasets used and/or analysed during the current study are available from the corresponding author on reasonable request.
