# Author Correction: *Saccharomyces cerevisiae* additions normalized hemocyte differential genes expression and regulated crayfish (*Procambarus clarkii*) oxidative damage under cadmium stress

**DOI:** 10.1038/s41598-023-48846-3

**Published:** 2023-12-15

**Authors:** Yaru Yang, Shuaidong Li, Yumin Zhu, Litao Che, Qifan Wu, Shijun Bai, Guocheng Shu, Xianming Zhao, Peng Guo, Salma A. Soaud, Nianzhen Li, Mengling Deng, Jia Li, Ahmed H. El-Sappah

**Affiliations:** 1 School of Agriculture, Forestry and Food Engineering, Yibin University, Yibin, 644000 China; 2 College of Morden Agriculture, Yibin Vocational and Technical College, Yibin, 644003 China; 3 Genetics Department, Faculty of Agriculture, Zagazig University, Zagazig, 44511 Egypt; 4 Faculty of Animal Science and Technology, Yunnan Agricultural University, Kunming, Yunnan 650201 China

Correction to: *Scientific Reports* 10.1038/s41598-023-47323-1, published online 28 November 2023

In the original version of this Article, Mengling Deng was incorrectly affiliated with affiliations 1, 2 and 3.

The correct affiliation is as follows:

Faculty of Animal Science and Technology, Yunnan Agricultural University, Kunming, Yunnan, 650201, China

The original Article has been corrected.

